# Pooled analysis of frontal lobe transcriptomic data identifies key mitophagy gene changes in Alzheimer's disease brain

**DOI:** 10.3389/fnagi.2023.1101216

**Published:** 2023-06-09

**Authors:** Taoyu Mei, Yuan Li, Anna Orduña Dolado, Zhiquan Li, Robin Andersson, Laura Berliocchi, Lene Juel Rasmussen

**Affiliations:** ^1^Center for Healthy Aging, Department of Cellular and Molecular Medicine, University of Copenhagen, Copenhagen, Denmark; ^2^Bioinformatics Centre, Department of Biology, University of Copenhagen, Copenhagen, Denmark; ^3^Department of Health Sciences, University Magna Græcia of Catanzaro, Catanzaro, Italy

**Keywords:** Alzheimer's disease, mitochondria, mitophagy, lysosome, transcriptomics, VCP, ARF1, GABARAPL1

## Abstract

**Background:**

The growing prevalence of Alzheimer's disease (AD) is becoming a global health challenge without effective treatments. Defective mitochondrial function and mitophagy have recently been suggested as etiological factors in AD, in association with abnormalities in components of the autophagic machinery like lysosomes and phagosomes. Several large transcriptomic studies have been performed on different brain regions from AD and healthy patients, and their data represent a vast source of important information that can be utilized to understand this condition. However, large integration analyses of these publicly available data, such as AD RNA-Seq data, are still missing. In addition, large-scale focused analysis on mitophagy, which seems to be relevant for the aetiology of the disease, has not yet been performed.

**Methods:**

In this study, publicly available raw RNA-Seq data generated from healthy control and sporadic AD post-mortem human samples of the brain frontal lobe were collected and integrated. Sex-specific differential expression analysis was performed on the combined data set after batch effect correction. From the resulting set of differentially expressed genes, candidate mitophagy-related genes were identified based on their known functional roles in mitophagy, the lysosome, or the phagosome, followed by Protein-Protein Interaction (PPI) and microRNA-mRNA network analysis. The expression changes of candidate genes were further validated in human skin fibroblast and induced pluripotent stem cells (iPSCs)-derived cortical neurons from AD patients and matching healthy controls.

**Results:**

From a large dataset (AD: 589; control: 246) based on three different datasets (i.e., ROSMAP, MSBB, & GSE110731), we identified 299 candidate mitophagy-related differentially expressed genes (DEG) in sporadic AD patients (male: 195, female: 188). Among these, the AAA ATPase VCP, the GTPase ARF1, the autophagic vesicle forming protein GABARAPL1 and the cytoskeleton protein actin beta ACTB were selected based on network degrees and existing literature. Changes in their expression were further validated in AD-relevant human *in vitro* models, which confirmed their down-regulation in AD conditions.

**Conclusion:**

Through the joint analysis of multiple publicly available data sets, we identify four differentially expressed key mitophagy-related genes potentially relevant for the pathogenesis of sporadic AD. Changes in expression of these four genes were validated using two AD-relevant human *in vitro* models, primary human fibroblasts and iPSC-derived neurons. Our results provide foundation for further investigation of these genes as potential biomarkers or disease-modifying pharmacological targets.

## 1. Introduction

Recent years have seen a growing trend in the number of Alzheimer's Disease (AD) patients, which reached 44 million in 2014 and 47 million in 2016 (Prince et al., 2014, 2016). This number is expected to increase to over 74 million by 2030 and 131 million by 2050 (Prince et al., 2015), causing significant burden on medical systems due to patients' need of long-term care (Prince et al., 2016). Characterized by brain atrophy and the accumulation of amyloid β plaques and neurofibrillary tangles in brain (Alzheimer, 1906), the aetiology of AD remains largely unknown. Effective treatments for AD are still lacking after decades of extensive research and investments.

Recent studies indicated that some AD pathological features may stem from defective disposal of dysfunctional mitochondria by mitophagy. For instance, inhibition of amyloid β aggregates and tau tangles, as well as restoration of cognitive ability, can be achieved through mitophagy inducers in C. elegans and AD mice models (Fang et al., 2019). Evidence suggests that compromised mitophagy in AD may be attributed to abnormalities in lysosomal activity and failure to incorporate dysfunctional mitochondria into lysosomes for the correct formation of autophagosomes (Kerr et al., 2017). Thus, lysosome and phagosome abnormality may be contributing factors to mitophagy dysfunction in AD. However, the molecular mechanisms underlying the disposal of defective mitochondria by the autophagic machinery and mitophagy malfunction in AD patients are yet to be systematically examined. Therefore, an investigation of mitochondria-centered mechanisms holds the promise to identify important molecular steps in the initiation and progression of the disease, as well as potential novel biomarkers and targets for intervention.

Meta-analysis of AD brain transcriptomic data (Patel et al., 2019) has shown that differential gene expression in the frontal, temporal, parietal lobe, and the cerebellum parallels the AD histopathological changes that differentially affect these brain regions in respect to both staging (Braak and Braak, 1991) and severity. Considerable differences in AD pathology and prevalence have also been observed between men and women (Grimm et al., 2016; Podcasy and Epperson, 2016). These important observations provided the scientific rationale for our investigation of AD gene changes separately in different brain regions and in both sexes.

Transcriptomic assays such as microarray and RNA-Seq have been widely used to investigate the gene expression profile behind the molecular mechanisms of AD in different brain regions. These have generated a considerable amount of data that have been made available through both general and AD-dedicated databases such as Accelerating Medicines Partnership-Alzheimer's Disease (AMP-AD) (Hodes and Buckholtz, 2016) and many genes and pathways that may play crucial roles in AD have been identified [e.g., YAP1 (Xu et al., 2018) & SPCS1 (Patel et al., 2019)]. Although these data have been thoroughly analysed, only few analyses focused on the role of mitochondria-related genes and pathways in AD. Moreover, RNA-Seq data provide chances to evaluate more genes that are not included or difficult to be detected by microarray analysis.

In fact, the results of many transcriptomic studies (especially microarray) suffer from lack of reproducibility and high variability (Zhang et al., 2009; Perng and Aslibekyan, 2020), partially due to limitations in sample sizes. Integrative analysis of publicly available datasets can be a solution to this problem while avoiding the expense of new costly high-throughput experiments. Possible methods to integrate datasets include: (1) merging of expression matrices, (2) meta-analysis of Differentially Expressed Gene (DEG) lists and (3) pooling together the raw data. While merging of expression matrices followed by cross-platform normalisation could be the only choice for microarray data from various platforms, to avoid batch effect and high false discover rate of meta-analysis, pooling of raw data at the beginning of analysis is more suitable and reliable for RNA-Seq data when the datasets are generated from similar samples and experimental settings. Though more popular in clinical research, pooled analysis has also been used for omics data analysis (Sonnenblick et al., 2018; Matikas et al., 2019; Traylor et al., 2021).

Network-based approaches are useful for identifying potential core genes among the DEGs. For instance, in Protein-Protein Interaction (PPI) networks, a gene whose product interacts with many other gene products (i.e., with higher degree in the graph) is in general more likely to be a key gene. At the same time, a gene targeted by more microRNAs that are regulated in the opposite direction to this gene, could be regarded to have more reliable differential expression, because a microRNA can bind to the mRNAs of its target gene and degrade the latter. Many unbiased screenings have been published, utilising module detection algorithms to these networks followed by enrichment analysis based on gene sets, or key genes determination for each module (Hu et al., 2020; Wan et al., 2020; Yu et al., 2021). In our opinion, it is more reliable to combine algorithms and the existing knowledge of a disease to avoid the ignorance about important key genes (especially those with smaller fold changes) that are related to particular biological processes, which are not necessarily enriched.

To the best of our knowledge, there is no integration study using the pooling method on RNA-Seq datasets from post-mortem brain samples of AD patients. Only two microarray-based integration studies have been published, using meta-analysis (Patel et al., 2019), and merging of expression matrices (cross-platform normalisation) (Xu et al., 2018), respectively. Other similar bioinformatics studies lack experimental validation or did not focus on mitophagy (Hu et al., 2020; Yu et al., 2021).

In this study, raw RNA-Seq data of brain AD samples and age-matched controls were acquired from publicly available sources. DEGs were identified after pooling together samples from the frontal lobe (FL) separately for each sex and correcting covariates and batch effects. Gene sets, whose descriptions include one of the three keywords (i.e., mitophagy, phagosome & lysosome), were collected from different databases (e.g., KEGG, GO, Mizushima, & Reactome). Their member DEGs were extracted for following Protein-Protein Interaction (PPI) network analysis via STRING. Four candidate genes (VCP, ARF1, GABARAPL1, and ACTB) were selected from highly connected genes in the PPI network from the STRING database (Szklarczyk et al., 2019) and the microRNA-mRNA interaction network for experimental verification. These genes were validated by literature search and experiments in state-of-the-art human AD models (skin fibroblast and iPSC-derived neurons). This research highlights four novel genes whose products and functions might be relevant for mitophagy and mitochondria alterations described in AD.

## 2. Materials and methods

### 2.1. Data collection

**Figure 1:**
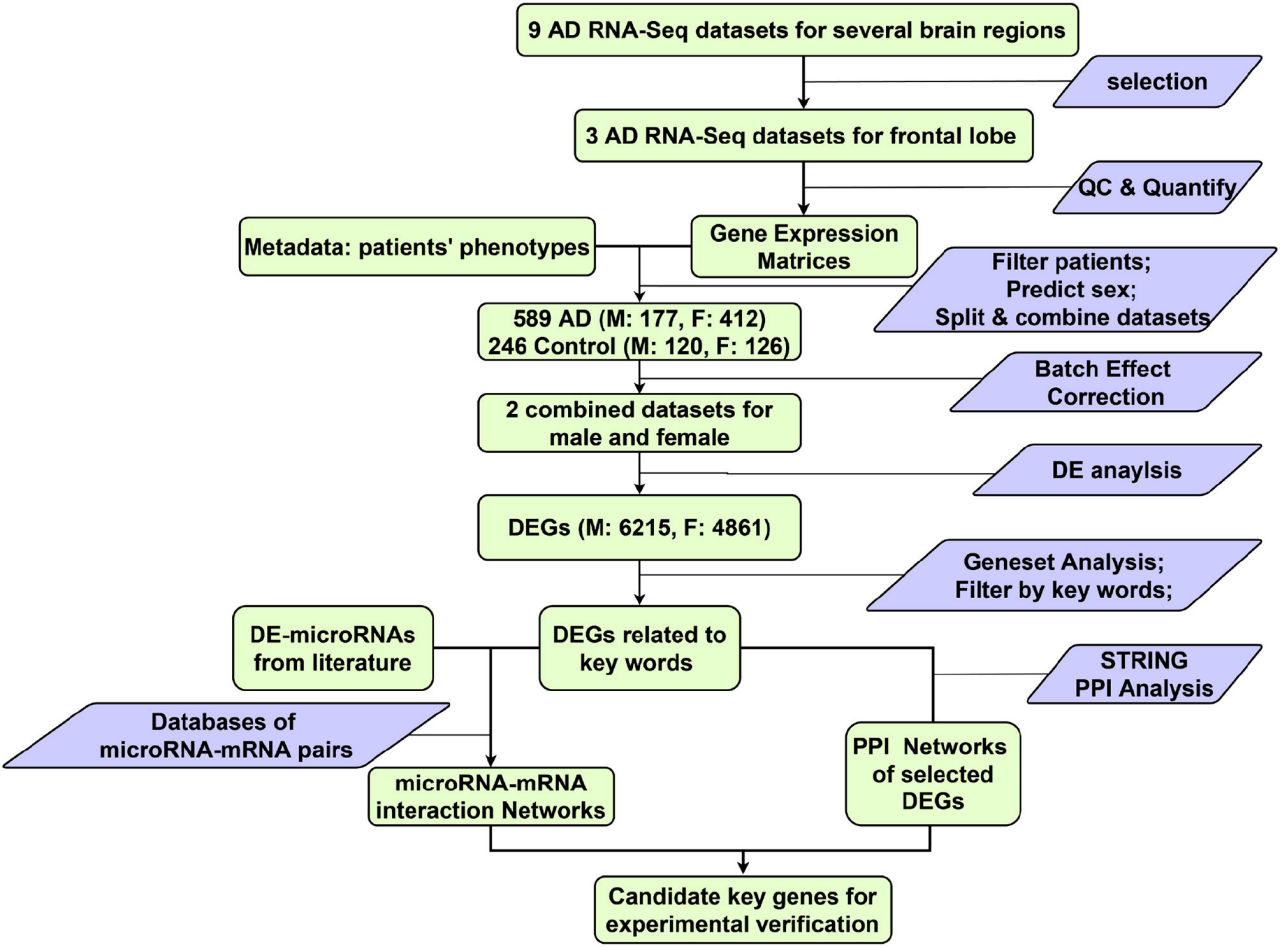
Overview of the work flow. Green rectangle, data; Blue parallelogram, operations on data; M, male; F, female.

The workflow of this study is shown in Figure 1. At the beginning, nine possibly eligible AD RNA-Seq datasets were identified: six GSE datasets downloaded from Gene Expression Omnibus (GEO) (https://www.ncbi.nlm.nih.gov/gds) and three AMP-AD datasets from several brain regions.

For the six GSE datasets (i.e., GSE125583, GSE125050, GSE110731, GSE104704, GSE95587, & GSE53697) (Scheckel et al., 2016; Friedman et al., 2018; Nativio et al., 2018; Li et al., 2019; Srinivasan et al., 2020), metadata describing patients' phenotype (e.g. age, sex, & Braak score) were collected from the GEO using R package GEOquery (v 2.48.0) (Davis and Meltzer, 2007). The raw RNA-Seq data were downloaded from the Sequence Read Archive (SRA) (https://www.ncbi.nlm.nih.gov/sra/) and converted to fastq format using fasterq-dump in SRAtoolkit (v 2.10.2).

For the three AMP-AD datasets (i.e., MayoRNAseq, MSBB, & ROSMAP) (Allen et al., 2016; De Jager et al., 2018; Wang et al., 2018), the metadata and RNA-Seq data in fastq format were both requested and downloaded from the Synapse (https://www.synapse.org/) via the Python package synapseclient and synapseutils. Instead of the BAM files stored in each study, fastq files generated by the RNAseq Reprocessing Study carried out by the AMP-AD consortium were used.

### 2.2. Inclusion criteria for datasets and patients

Datasets were only included in the analysis if they were generated by a study that (1) utilised bulk RNA-Seq; (2) included at least 5 AD patients and 5 age-matched controls; and (3) examined post-mortem samples of human brain. Eligibility criteria further required patients to be aged above 65 to ensure only late-onset AD patients were considered. The Braak score (Braak et al., 2006) was recorded in the metadata of most datasets, and was used as the diagnosis criteria of AD. Patients with Braak score 0, I, or II were classified into control groups, while IV, V, VI were classified into AD groups. Patients with a Braak score of III were excluded due to ambiguity in AD diagnosis.

### 2.3. Quality control and preprocessing of RNA-Seq data

An overview of the sequencing quality was visualised for each sample by FASTQC (v 0.11.9) (Andrews, 2010) and summarised by each dataset by using R package ngsReports (v 1.2.0) (Ward et al., 2020). Trimmomatic (v 0.39) (Bolger et al., 2014) was utilised to trim low-quality bases from reads and discard low quality reads from samples in the “sliding-windows” mode. Sequences originating from adaptors were also removed with Trimmomatic “palindrome” mode. Values of parameters were chosen partially based on observation and recommendations (MacManes, 2014).

Salmon (Patro et al., 2017) was adopted to quantify the transcript expression for all samples, using human reference transcriptome and annotations from GENCODE (Frankish et al., 2019) (release 33). A shell script “shuffle.sh” in BBmap (Bushnell, 2014) was applied to randomise the reads in these fastq files while keeping each pair of reads for paired-end (PE) data at the same place in the pair of fastq files. The expression values at transcript level were then summarised to gene level by applying R package tximport (v 1.14.0) (Soneson et al., 2016).

To include samples without sex information in the metadata for the following analyses and to avoid mislabeling, prediction of sex was performed on all samples. The prediction was based on a ratio between total read counts on chromosome Y and total read counts on chromosome X as well as Principal Component Analysis (PCA).

### 2.4. Integrating datasets and differential expression analysis

For the three datasets (i.e., ROSMAP, MSBB, & GSE110731) of Frontal Lobe (FL) shown in Table 1, age and Post-Mortem Interval (PMI) were included as covariates. Surrogate Variables (SVs) were then estimated for variance in gene expression that cannot be explained by the two known covariates using R package SVA (v 3.28.0) (Leek, 2014).

**Table 1:** Three selected AD frontal lobe RNA-Seq datasets.

**Database**	**Dataset**	**Published year**	**Brain region**	**Sample size**	
				**AD (M / F)**	**Control (M / F)**
AMP-AD	MSBB	2015	Frontal lobe	415 (132 / 283)	137 (63 / 74)
AMP-AD	ROSMAP	2015	Frontal lobe	505 (147 / 358)	153 (74 / 79)
GEO	GSE110731	2019	Frontal lobe	10 (6 / 4)	10 (5 / 5)
/	Total (before filtering)	/	/	930 (285 / 645)	300 (142 / 158)
/	Total (after filtering)	/	/	589 (177 / 412)	246 (120 / 126)

M, male; F, female.

To visualise the effectiveness of batch effect correction, PCA was applied before and after batch correction. Variance stabilising transformation was employed using R package DESeq2 (v 1.22.2) (Love et al., 2014). The “removeBatchEffect” function in R package limma (v 3.36.5) (Ritchie et al., 2015) was then utilised to adjust the gene expression matrices by the covariates including SVs. Adjusted gene expression matrices were only used for visualisation, not DE analysis.

Mapping of detailed brain regions to broader brain regions was carried out partially according to the criteria used by two previous studies (Xu et al., 2018; Patel et al., 2019). For example, frontal cortex, superior frontal gyrus, and dorsolateral prefrontal cortex were all mapped to frontal lobe.

DE analysis was performed using R package DESeq2 (v 1.22.2) (Love et al., 2014). Briefly, raw count matrices, metadata (including covariates & disease status) and average transcript lengths were directly passed to DESeq2 which calculated normalisation factors for each sample (Anders and Huber, 2010) based on the average transcript length estimated by the R package tximport (v 1.14.0) (Soneson et al., 2016). Covariates were also included in the modelling process to correct for batch effects. The resulting p-values were adjusted using Benjamini-Hochberg method (Benjamini and Hochberg, 1995) for multiple testing to control for False Discovery Rate. Genes with an adjusted *p*-value < 0.05 and an absolute value of log2 fold change >0.1 were considered significant DEGs for downstream analysis. Known AD-related genes from a systematic review (Hu et al., 2017) were used to validate the DE analysis results.

### 2.5. Selection of genes related to mitophagy

To extract DEGs between healthy control and sporadic AD patients related to mitophagy, several geneset databases were utilised, including wikiPathways (Slenter et al., 2018) (v 20200510), GO (Ashburner et al., 2000; The Gene Ontology Consortium, 2019), KEGG pathway (Kanehisa and Goto, 2000), KEGG module, Reactome (Jassal et al., 2019), and MSigDb (Liberzon et al., 2011, 2015). Although MSigDb includes GO, KEGG and Reactome, the latter 3 databases were still included separately to secure the latest versions. Redundant genes were removed. Genesets whose names contained at least one of the three keywords (i.e., mitophagy, lysosome & phagosome) were collected. Their member genes were extracted using R package clusterProfiler (v 3.8.1) (Yu et al., 2012) and combined into a union of non-redundant mitophagy-related genes. The intersection between mitophagy genes and DEGs were passed on to downstream analyses.

### 2.6. Network analysis and candidate key gene selection

To construct PPI networks, the STRING database (v 11) (Szklarczyk et al., 2019) was adopted to extract known interactions between mitophagy-related DEGs. For each sex, the above selected DEGs (refer to 2.5) were uploaded to STRING via its API using R (v 3.6.1). Among DEGs that were not selected (i.e., differentially expressed but not a member gene of the above genesets), those interacting with at least 20 selected DEGs were also uploaded, because they might also be closely related to mitophagy. The degree and log 2 fold change of genes were visualised by the sizes and colours of nodes in the PPI plot respectively.

microRNA-mRNA pairs were downloaded from 2 databases: mirTarBase (Chou et al., 2018) and miRSponge (Wang et al., 2015), with the ID being unified according to miRBase, while differentially expressed microRNA in AD frontal lobe were obtained from a systematic review (Takousis et al., 2019) which combined RNA-Seq and microarray results regardless of male or female. A microRNA-mRNA interaction network was built based on these data.

Criteria for selecting key genes from down-regulated genes were: (1) degree ≥ 20 in male or female PPIs; (2) targeted by at least 10 up-regulated microRNAs; (3) the number of down-regulated microRNA targeting the gene should not exceed 1/3 of that of up-regulated microRNA; (4) has at least some implication in AD or mitophagy or mitochondria in literature.

### 2.7. Primary cells and culture condition

Primary skin fibroblasts [AG02261 (Ctrl1), AG16086 (Ctrl2), AG07377 (AD1), and AG06263 (AD2)] were obtained from the Coriell Institute for Medical Research (NJ, USA). Two fibroblast cell lines from sporadic AD (sAD) patients and two correspondent healthy sex- and age-matched samples have been used (see Supplementary Table 1 for details about age, sex, and disease status). Human fibroblasts were cultured in AmnioMAX^TM^-II Complete Medium (Gibco, #11269016) in 5% *CO*_2_ in a humid incubator at 37°C.

### 2.8. iPSCs information and culture condition

iPSCs were obtained from Zameel Cader at Nuffield department of Clinical Neurosciences, University of Oxford. Three iPSCs from sAD patients and three corresponding healthy sex- and age-matched samples were used (see Supplementary Table 2 for details about age and sex of the patients). iPSCs were cultured in Matrigel-coated plates and cultured with mTesR1 medium.

### 2.9. iPSCs-derived neurons

iPSCs were differentiated into neurons following a previously validated and published protocol (Zhang et al., 2013). Briefly, iPSCs were cultured in mTesR1 medium added with Ngn2 and rtTA lentivirus on day -1, and then the next day (Day 0) the culture medium was replaced with DMEM/F12 medium containing N2 (Thermo Fisher), NEAA (Invitrogen), human BDNF (10 mg/l, PeproTech), and human NT-3 (10 mg/l, PeproTech). Doxycycline (2 g/l, Clontech) was added on day 0 to induce TetO gene expression. On day 2, a 24 hr puromycin (1 mg/l) selection period was started. On day 3, medium was changed to Neurobasal medium containing B27(Thermo Fisher), Glutamax (Invitrogen), BDNF and NT3; After day 7, 50% of the medium in each well was exchanged every 2 days. On day 14, iPSCs-derived neurons (iN) were collected and processed for RNA extraction and gene expression analysis.

### 2.10. RNA extraction and gene expression analysis

Total RNA was isolated from both fibroblast and induced neurons (iN), using RNeasy MiniKit (Qiagen) following the manufacturer instruction. RNA was analysed both for purity and concentration with an Agilent BioTek spectrometer (CHECK). Samples with OD260/280 ratio around 2.0 were included in the gene expression analysis. cDNA was synthesized using FIREScript RT cDNA synthesis MIX with oligo (dT) primer (Solis BioDyne) according to the manufacturer instructions. Real-time (RT)-PCR was performed using HOT FIREPol EvaGreen qPCR mix from Solis BioDyne following instructions from manufacturer using an Applied Biosystems QuantStudio 6Flex Real-Time PCR system. Three housekeeping genes, HPRT1, RPL13, and TBP were tested for their suitability as control genes.HPRT1 and RPL13a have already been shown to have a stable expression in several brain diseases (Penna et al., 2011; Rydbirk et al., 2016; Panina et al., 2018; González-Bermúdez et al., 2019). Also in our models, they showed ubiquitous gene expression both across brain regions and reprogrammed iPSCs and were therefore chosen as reference genes for normalisation in the gene expression analysis. The relative mRNA expression was calculated using the 2^−ΔΔCT^ method. Expression analysis of the following genes was undertaken: ARF1, ACTB, GABARAPL1, GAPDH, and VCP. All oligonucleotide primers were designed using PrimerBlast (NCBI-NIH) and analyzed with NetPrimer (BioSoft) (Supplementary Table 3).

### 2.11. Statistics

Statistical analysis was performed with Prism 6.0 (GraphPad Software, https://www.graphpad.com/scientific-software/prism/). One-way multiple comparisons Student's *t*-test with Bonferroni's multiple comparison *post hoc* test was used for mean comparisons between conditions as required. The number of samples used for statistical analyses (n) refers to either the number of different individuals per group or total samples in the study, being always four independent biological replicates. Differences were considered significant when *p* < 0.05. Data in bar graphs are reported as mean ± SD.

## 3. Results

### 3.1. Datasets selection

Three bulk RNA-Seq datasets of post-mortem frontal lobe samples from AD patients and age-matched controls were selected (Table 1). Datasets for other brain regions were excluded for incomplete metadata and low data quality. After combining the 3 datasets and filtering the samples by the inclusion criteria (refer to 2.2), a total of 589 AD (M: 177; F: 412) and 246 control (M: 120; F: 126) frontal lobe (FL) samples were analysed, following the workflow in Figure 1.

### 3.2. DE analyses based on integrated data

**Figure 2:**
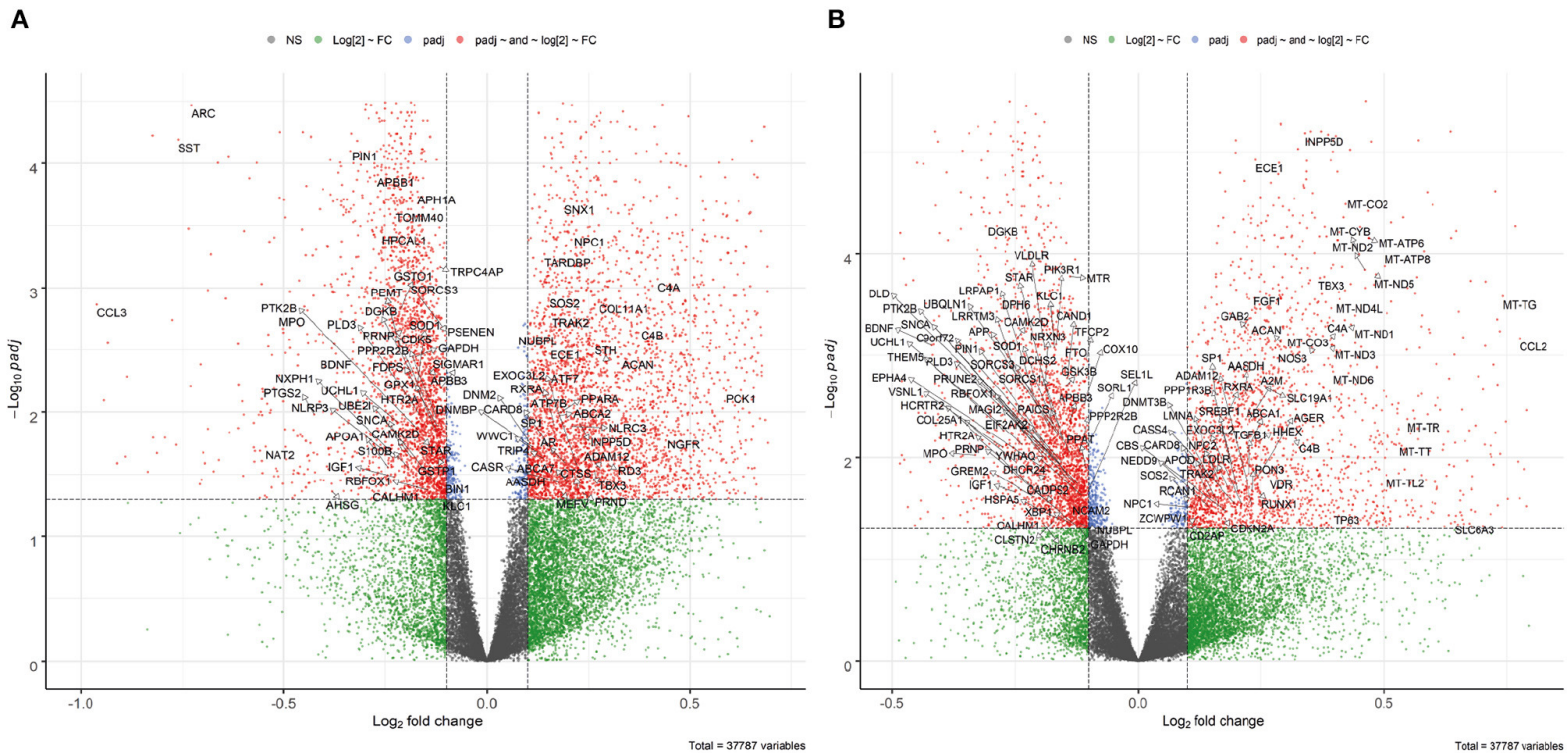
Volcano plot of DE analysis for the combined FL dataset. Significant DEGs are shown in red points, with known AD-related genes highlighted. **(A)** Male: 3,548 up, 2,667 down. **(B)** Female: 2,299 up, 2,562 down.

For the combined FL datasets, the expression levels of 37,787 genes (incl. protein-coding genes and non-coding RNAs) were quantified. The Volcano plots of the combined dataset show that most of the significant DEGs have small absolute values of log2 fold change which may be due to variation in gene expression between samples and datasets (Figure 2). It is noted that smaller effect sizes are common phenomena for integrative studies (Xu et al., 2018; Patel et al., 2019) compared to what is generally observed for DE analysis of single datasets.

In total, 4,457 male specific, 3,103 female specific, and 1758 shared AD DEGs were identified in the frontal lobe. Many genes have been previously reported as AD-associated (Hu et al., 2017), whose gene symbols are shown in Figure 2. For instance, brain-derived neurotrophic factor (BDNF) was down-regulated in both sexes, consistent with previous findings that a reduction in BDNF is associated with cognitive impairment in AD (Amidfar et al., 2020), whereas elevated BDNF levels can improve cognitive function in an AD mouse model (Choi et al., 2018). Another example is the transcription factor SP1 (specificity protein 1) whose up-regulation was observed in both sexes, in line with previous studies in an AD mouse model (Citron et al., 2015), as well as in human AD cortex and hippocampus (Villa et al., 2013). Such reference genes served as a validation of the DE analysis.

### 3.3. Genesets related to keywords and their member DEGs

Fifty-three genesets containing the key words mitophagy, lysosome or phagosome were identified in 4 databases (Table 2). No relevant genesets were found in the wikiPathways database, which is thus not shown in the table. The Mizushima database is a part of MSigDb. Some well-studied mitophagy pathways are included in the above genesets, such as receptor-mediated mitophagy (Yamaguchi et al., 2016) and PINK1/PARKIN-mediated mitophagy (Eiyama and Okamoto, 2015). Both functional and morphological genesets were included in the analysis. Taking the union of their member genes, 716 non-redundant genes were regarded as mitophagy-related, among which 195 were differentially expressed for males, 188 for females, and were further used for network analysis.

**Table 2:** Number of genesets potentially related to mitophagy in each database.

**Database**	**Keyword**	**Number of gene set**
KEGG	Mitophagy	KEGG mitophagy animal
	Lysosome	KEGG lysosome
	Phagosome	KEGG phagosome
GO	Mitophagy	5, e.g., GO mitophagy, GO negative regulation of mitophagy
	Lysosome	25, e.g., GO endolysosome, GO golgi to lysosome transport
	Phagosome	15, e.g., GO phagosome acidification, GO phagosome maturation
Reactome	Mitophagy	3, e.g., REACTOME receptor mediated mitophagy, REACTOME pink parkin mediated mitophagy
	Lysosome	REACTOME lysosome vesicle biogenesis, REACTOME prevention of phagosomal lysosomal fusion
	Phagosome	REACTOME cross presentation of particulate exogenous antigens phagosomes, REACTOME suppression of phagosomal maturation
Mizushima	Phagosome	MIZUSHIMA autophagosome formation

### 3.4. Network analysis: PPI and microRNA-mRNA network

In order to identify potential key genes among numerous relevant DEGs, 2 PPI networks were obtained from the STRING database for male and female, respectively. In the networks, each node represents a DEGs. DEGs that were not member of relevant genesets but interacted with at least 20 members of the network were also added to the network (shown in rectangles instead of circles) and defined as candidate new members of these genesets.

**Figure 3:**
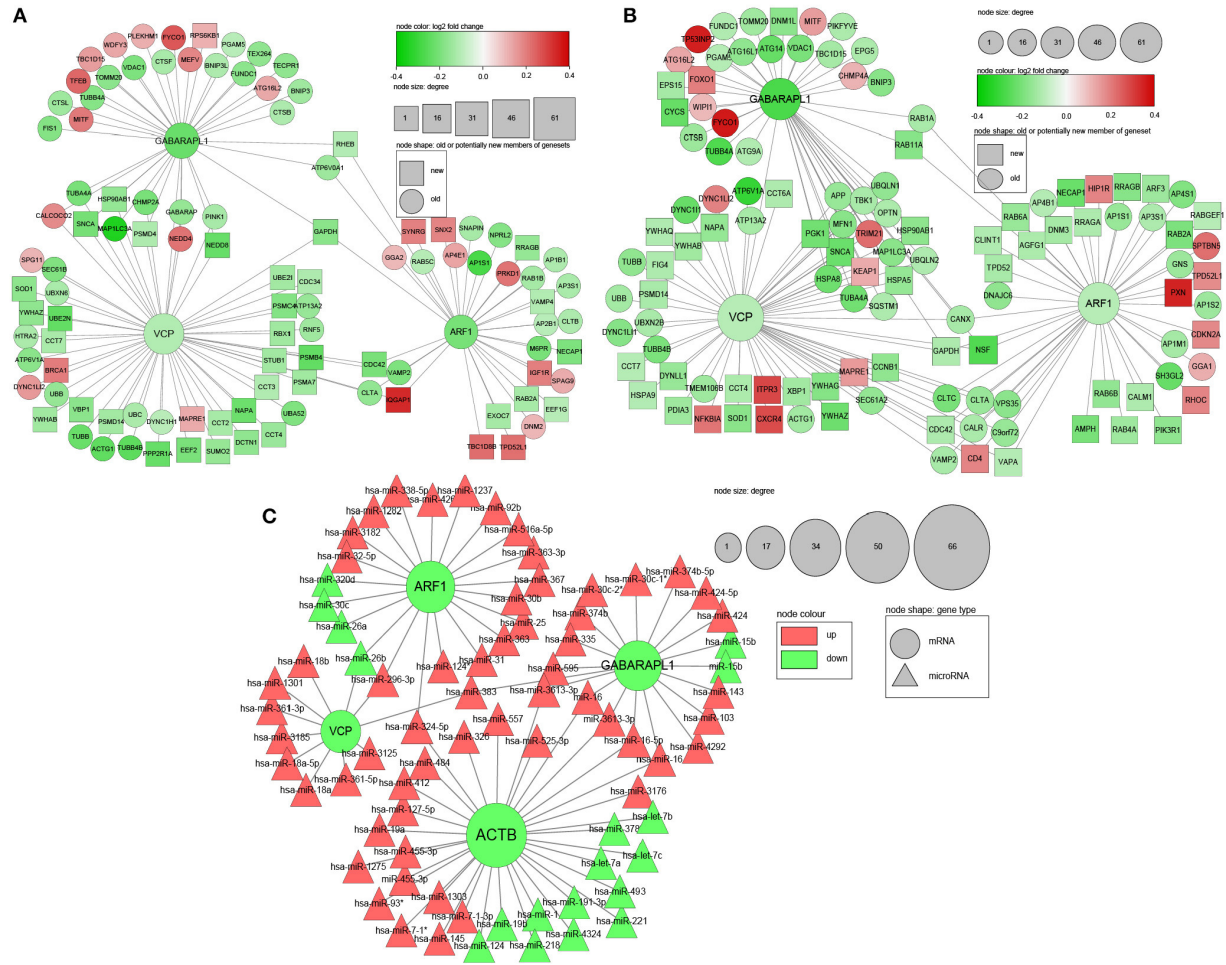
PPI and microRNA-mRNA interaction networks of DEGs related to mitophagy in frontal lobe (FL). **(A)** PPI of male DEGs; **(B)** PPI of female DEGs; **(C)** microRNA-mRNA interaction network based on selected common DEGs between male and female, as well as microRNA-mRNA pairs without sex information. Red: up-regulated genes; Green: down-regulated genes; Circle: genes that are already included in genesets related to mitophagy; Rectangle: candidate new member genes of geneset related to mitophagy; Triangle: microRNA; Node sizes are in proportion to node degree; Depth of node colours are in proportion to log2 fold change.

The size of each node is proportional to its degree (the number of genes that a certain gene interacts with). The direction of gene regulation is indicated by the gene nodes' colour, with down-regulated genes in green while up-regulated genes in red. The depth of a node's colour is in proportion to the absolute value of the log2 fold change of a gene, which ranges from 0 to 0.4 (Figure 3).

The PPI network construction began with 195 genes for male and 188 genes for female. After adding candidate new members, the total number of nodes were 311 for male and 298 for female. In total of 3,641 and 3,677 protein interactions for male and female were retrieved from the STRING database respectively, forming the edges of the PPI network.

MicroRNAs' suppression of their target mRNAs was regarded as evidence for genes' differential expression at mRNA level. From literature and databases, 559 unique DE-microRNA in FL (395 up, 128 down), and 380,639 microRNA-mRNA pairs were retrieved to create the microRNA-mRNA interaction network. MicroRNAs that were reported in some papers as up-regulated in AD FL but also reported to be down-regulated in the same number of studies were excluded from the analysis.

### 3.5. Genes selection for experimental validation

Based on the aforementioned criteria (refer to 2.6) and existing knowledge, four genes were finally selected: VCP, ARF1, GABARAPL1, and ACTB (Table 3), which are highly connected in both PPI (Figures 3A, B) and microRNA-mRNA network (Figure 3C). For brevity, only the first three genes and genes/microRNAs that interact with them are shown in the figure. The network visualisation was done in Cytoscape (v 3.8) (Shannon, 2003).

**Table 3:** Genes selected for experimental validation.

**Gene**	**Description**	**Log2 fold change**		**Degree in PPI**		**Number of targeting microRNA**	
		**Male**	**Female**	**Male**	**Female**	**Up**	**Down**
VCP	Valosin containing protein	–0.11	–0.10	56	61	10	1
ARF1	ADP ribosylation factor 1	–0.21	–0.10	33	46	17	4
GABARAPL1	GABA type A receptor associated protein like 1	–0.23	–0.28	37	45	17	2
ACTB	Actin beta	–0.17	–0.05 (n.s.)	29	/	22	12

As expected, a number of known mitophagy genes interact with the selected genes. For example, FUN14 domain containing 1 (FUNDC1), which interacts with GABARAPL1 and is down-regulated in both sexes, assists the phagophore to engulf dysfunctional mitochondria for mitophagy (Cai and Jeong, 2020). VDAC1 and OPTN are known mitophagy mediators as well (Bakula and Scheibye-Knudsen, 2020). Interestingly, β-Synuclein (SNCA), which is not a member of any canonical mitophagy genesets/pathways, is shown to interact with many mitochondrial outer membrane components and play a role in mitochondrial dysfunction (Malpartida et al., 2021) and mitophagy (Shaltouki et al., 2018) in Parkinson's disease. It also interacts with VCP and GABARAPL1 in the PPI network in this study. Glyceraldehyde 3-phosphate dehydrogenase (GAPDH), also not a member of any canonical mitophagy genesets/pathways, promotes the fusion of damaged mitochondria with lysosome, thus promoting mitophagy in Huntington's disease (Hwang et al., 2015). It interacts with the three selected genes. In summary, the above evidence supports the reliability of the PPI networks in this research.

In the microRNA-mRNA interaction network (Figure 3C), most of the microRNAs targeting the first three selected down-regulated genes are up-regulated, supporting the differential expression of these genes. For microRNAs targeting ACTB, there are still more up-regulated than down-regulated microRNAs, though the numbers are close.

### 3.6. Experimental validation of VCP, ARF1, GABARAPL1, and ACTB

**Figure 4:**
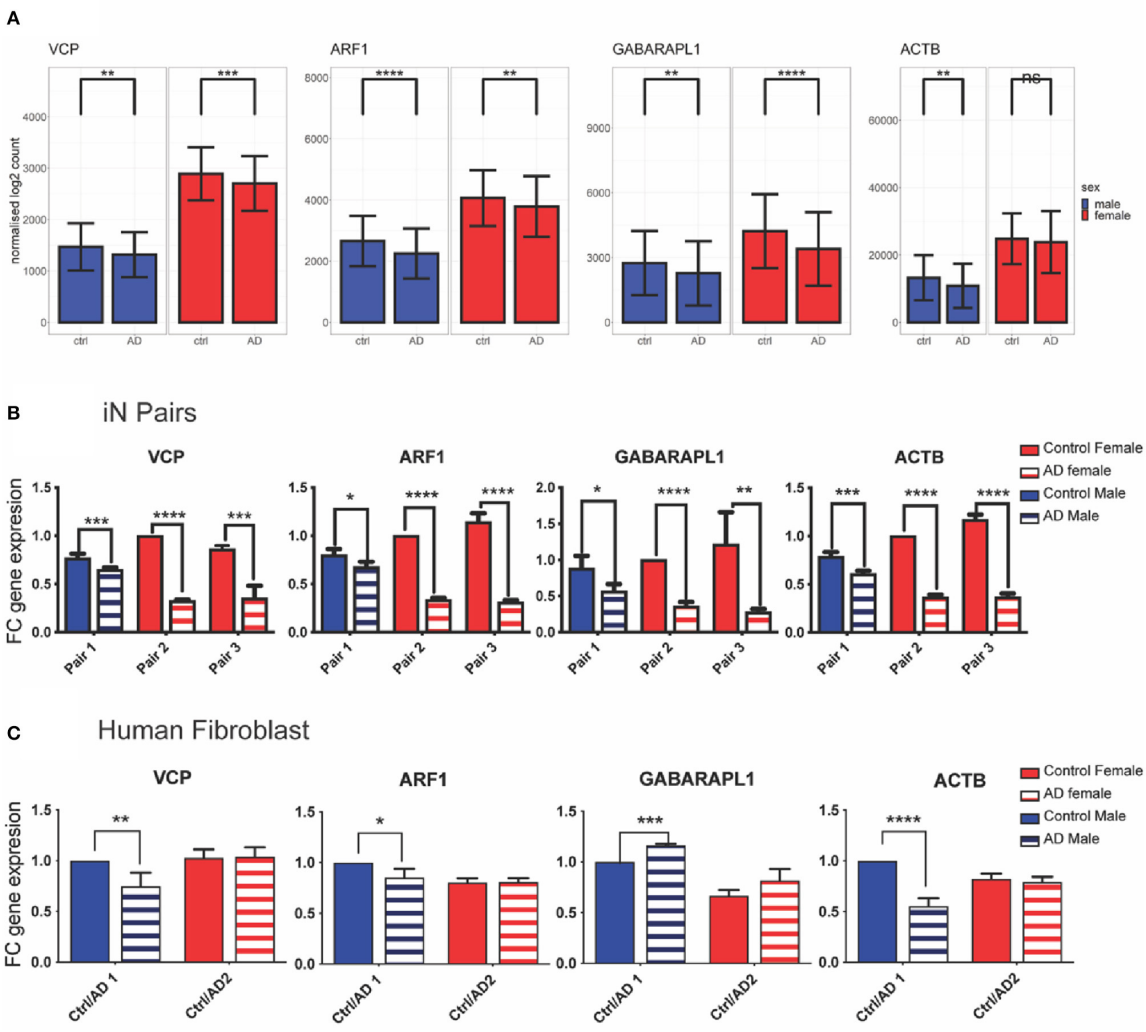
Experimental validation of differential expression gene analysis in preclinical AD models. **(A)** Bioinformatic analysis of differential gene expression; **(B)** Individual gene expression in iN samples paired samples by age and gender (total *n* = 6; Multiple pairwise *t*-test); **(C)** Individual gene expression in human fibroblast samples paired by age and gender (total *n* = 4; Multiple pairwise *t*-test). Solid graph bars represent control groups and striped bars represent AD groups; blue and red bars represent male and female individuals, respectively. Data in graph bars are reported as mean with their corresponding standard deviation. Statistical significance is reported as *p < 0.05; **p < 0.01; ***p < 0.005 and ****p < 0.0001.

The average expression levels of the four selected genes in the combined AD FL transcriptomics dataset is shown in Figure 4A. To validate the bioinformatic results, the expression of the four down-regulated genes related to mitophagy in Alzheimer's disease, which had been selected (i.e., ARF1, GABARAPL1, VCP, and ACTB) were analysed by qPCR in AD-relevant cellular models. In iPSCs-induced neurons (iN), the expression of all the four genes was significantly decreased in sAD patients when compared to age and sex matched healthy controls. This was observed in both males and females but was more pronounced in females (Figure 4B). In the case of human fibroblasts, significant changes in the relative gene expression between AD patients and age/sex-matched healthy individuals was detected only in males but not in females. In particular, VCP, ARF1, and ACTB showed a significant reduction of expression, whereas GABARAPL1 was slightly increased in human fibroblast from male AD patients compared to healthy controls (Figure 4C). In the case of the female samples no differences in gene expression were detected between AD fibroblasts and matching controls in the case of VCP, ARF1, ACTB, or a slightly increased expression in the case of GABARAPL1 (Figure 4C), although not statistically significant.

## 4. Discussion

Prior studies have noted the essential role of mitochondria and mitophagy in AD, but the molecular mechanisms and relevance are still to be further investigated and understood (Kerr et al., 2017). Despite the large number of AD transcriptomics studies, performing sex- and brain region-specific integrative analysis on existing data, thus exploiting large sample sizes, and using genesets- and network-based approaches with a selective focus on specific biological processes such as mitophagy can lead to the identification of important key genes, which might have been previously ignored. Once experimentally validated, these genes could turn to be promising core genes in AD pathogenesis.

With the idea of optimizing the use of publicly available data, we pooled together three RNA-Seq datasets generated from frontal lobe samples of AD patients and healthy controls. We used them for DE analysis with batch effects correction (Figures 1, 2). Several DEGs were identified through geneset analysis, PPI, and microRNA-mRNA network analysis (Figure 3), which could be critical players in mitochondria-centered AD mechanisms. All these genes were related to at least one of the keywords: mitophagy, lysosome, and phagosome.

The prevalence of AD, as well as its incidence in the individual over 80, is significantly higher in women compared to men (Mielke et al., 2014). Recent epidemiological data suggest that women present faster cognitive decline than men (Levine et al., 2021), thus suggesting the existence of sex difference in AD risk factors, presentation, and progression. However, the molecular mechanisms of sex-biased differences in AD and the sex-specific effects of some genes in AD are still elusive (Dumitrescu et al., 2019; Guo et al., 2022). The use of publicly available datasets allows for large sample size and the possibility of comparing differences between sexes.

Here, we started from datasets containing complete metadata for each sample as well as large enough sample sizes, and patients that were unambiguously diagnosed with late onset AD.

As to quality control, in each sequencing read, bases with high probability of being sequencing errors, which are referred to as low quality bases, were removed. This also applies to technical sequences (e.g., adaptors) that were used to complete the sequencing process and did not contain biological information from the original RNA sequence (Bolger et al., 2014). After this, high quality sequencing reads from each sample were assigned to each transcript in the reference transcriptome, generally based on sequence similarity, in order to quantify the expression level of each gene (Patro et al., 2017).

The main brain area affected in AD, the cerebral cortex, is anatomically divided into four lobes: frontal, parietal, occipital, and temporal lobe. Each of the lobes is further organised in complex structural subregions, i.e., *gyri* (bumps) and *sulci* (groves or fissures), with specific functions. Since RNA-Seq data from small sub-regions of the cerebral cortex are not abundant, we considered sub-regions belonging to the same lobe as equivalent with regard to how their RNA-Seq data reflect AD pathology, as also previously reported (Xu et al., 2018; Patel et al., 2019). By integrating data at lobe level, much larger sample size can be achieved to enable novel discovery.

Even for the same brain region, RNA-Seq datasets generated by different laboratories usually show varying gene expression levels, possibly due to different tissue-sampling methods, sequencing operations or other technical factors specific to each laboratory (Lazar et al., 2013). These un-wanted non-biological variations (i.e., batch effect), which also occur between batches within the same dataset, can even conceal the differences between AD and control, necessitating batch effect correction when integrating datasets. Therefore, batch effect correction was performed on the selected datasets before conducting differential expression analysis (Figure 1).

Differential expression analysis is a statistical test with a null hypothesis that the log2 fold change between AD and control for a gene's expression level is zero. Since RNA-Seq's read counts do not follow normal distribution but negative binomial distribution instead, and there is an obvious dependency between mean and variance, *t-*test is not applicable here. We thus adopted Wald test and other necessary operations via the R package DESeq2 (Love et al., 2014).

From nine DEGs shortlisted by their PPI degrees and by the regulation directions of microRNAs targeting them, four genes were selected based on bibliographic research on their involvement in mitochondrial function and autophagy: ARF1 (ADP ribosylation factor 1), GABARAPL1 (GABA type A receptor associated protein like 1), VCP (valosin containing protein), and ACTB (actin beta). Among them, the ACTB product is a cytoskeletal protein with typically housekeeping functions and considered having a stable expression. However, actin and actin-binding proteins have also been recognised as histopathological structures, which may contribute to AD pathogenesis and to primary behavioural symptoms of the disease (Bamburg and Bloom, 2009). Indeed, dysregulation of actin cytoskeleton observed in mouse AD models has been related to synaptic impairment and dendritic spine loss associated to AD (Rush et al., 2018). and recent genomic convergence and network analysis have found ACTB to be a significant AD risk gene (Talwar et al., 2014). In our study, the bioinformatic analysis on human transcriptomic data highlighted a statistically significant decrease on ACTB expression in AD individuals compared to matching controls in males, but not in women (Figure 4A). In both human iN and fibroblasts, this trend was further confirmed in male samples, whereas a statistically significant ACTB downregulation was detected also in AD iN in females (Figures 4B, C).

Of the other three genes, the ATPase VCP has been previously described for its role in Parkin-dependent mitophagy by extraction of ubiquitinated proteins from the outer mitochondrial membrane and selective degradation of damaged mitochondria through autophagosome fusion (Tanaka et al., 2010; Xu et al., 2011; Papadopoulos and Meyer, 2017; Bento et al., 2018). Also in neuronal cells, mitochondrial function seems to depend on VCP-mediated quality control (Fang et al., 2015) and VCP has been shown to bind the ER-associated protein UBXD2, which accumulates in neurons of the AD brain at early stages (Liang et al., 2006). Furthermore, it has been recently reported that VCP mutations can be associated with tau pathology, thus supporting an important role of VCP in AD pathogenesis (Darwich et al., 2020).

Our results show in the bioinformatic analysis a significant decrease both in males and females (Figure 4A). Similarly, this reduced expression of VCP in male samples was also significant in iN and fibroblast whereas for females samples it only reach statistical significance in iN but not fibroblast (Figures 4B, C).

ARF1 is a small GTPase classically studied for its involvement in the trans-golgi vesicle transport (Donaldson and Jackson, 2011) and seems to play a role in the post-Golgi trafficking and recycling of BACE1, suggesting that alterations in ARF1 may perturb BACE1 traffic and increase the processing of Amyloid β precursor protein (APP) which generates more Aβ40 and Aβ42, the main pathogenic β-amyloid peptides and precursors of the amyloid plaques (Tan et al., 2020). Recent studies in cancer cell lines highlighted the important role of Arf-1 at the contact sites between ER and mitochondria, and how Arf-1 contributes to maintain mitochondrial morphology and function (Andersen et al., 2020). Interestingly, in yeast and C. elegans, Arf-1 has also been shown to regulate mitofusin/Fzo1 homeostasis and the removal of its toxic mitochondrial clusters (Ackema et al., 2014). In the present study the bioinformatic transcriptomics analysis highlighted a significant decrease in AD individuals compared to matching controls both in males and females (Figure 4A). This observation was further confirmed in iN from both male and female AD samples (Figure 4B), whereas in fibroblasts a statistically significant downregulation of ARF-1 was detected only in male AD patients (Figure 4C). Previous expression profiling studies indicate that Arf-1 is likely to act in most if not all tissues, but given the different membrane trafficking requirements of different cell types it seems possible that its gene expression varies between cell types (Chintapalli et al., 2007).

GABARAPL1 is a member of the LC3/GABARAP protein family and ortholog of ATG8. It mediates receptors trafficking to the plasma membrane (Schaaf et al., 2016) and participates to autophagosome formation by interacting with Nix and regulating the pool of healthy mitochondria (Novak et al., 2010). Under mitochondrial stress conditions, GABARAPL1 has been shown to promote the clearance of damaged mitochondria (Novak et al., 2010), whereas its decreased expression has been associated to accumulation of the damaged organelles (Boyer-Guittaut et al., 2014; Li et al., 2020). Interestingly, GABARAPL1 appears to be more highly expressed in the CNS as compared to other family members (Grand et al., 2013), however its role so far has been mainly investigated in antimicrobial responses (Sasai et al., 2017) and cancer (Boyer-Guittaut et al., 2014). Only very recently, a bioinformatic analysis reported GABARAPL1 as one of the genes differentially expressed in peripheral blood of AD patients (Wang et al., 2022), whereas proteome-wide analysis of brain extracellular vesicles seems to suggest that GABARAP proteins can be actively incorporated in these vesicles and this mechanism may be disrupted with AD progression (Gallart-Palau et al., 2020). Here, we describe for the first time a decreased GABARAPL1 expression in iPSCs-derived neurons from AD patients compared to healthy individuals, both in males and females (Figure 4B). On the contrary, a slight increase was observed in primary fibroblasts, which was statistically significant only in the male samples (Figure 4C). While GABARAPL1 is present at comparable mRNA levels in all fetal tissues, it seems to be differentially expressed in adult tissues with the highest expression levels observed in the brain, heart, liver, skeletal muscle, kidney, spleen, ovary, small intestine, placenta, and peripheral blood leukocytes (Le Grand et al., 2011). The different trend observed in GABARAPL1 gene expression between the two cell types highlights the importance of comparing different cellular models when investigating potential disease biomarkers. In this study, the results obtained in iN, a cellular system in line with the human brain samples, validated the bioinformatics data for all the four selected genes and could be used as a reference system.

Changes in expression of the four genes (ACTB, VCP, ARF1 GABARAPL1) here identified by the bioinformatic analysis were further validated in two state-of-the-art models in the field of AD research, and in translational research aimed to target discovery and drug development for this neurodegenerative disease (de Leeuw and Tackenberg, 2019). Neurons derived by IPSC of patients are widely used as they have been shown to recapitulate key aspects of AD, both for the sporadic and familial form. Furthermore, the directed differentiation into a single cell type culture allows to study a cell type-specific phenotype. However, one general concern is lack of maturity and aging signatures of iPSC-derived neural cells, due their reprogramming (de Leeuw and Tackenberg, 2019). For this reason, in this work gene expression was validated also in human primary skin fibroblast from patients. In fact, the use of skin fibroblasts from patients represents an exceptional complementary tool for *in vitro* investigations as they appear to recapitulate early pathological events shown in the AD brain (Olesen et al., 2022). Finally, it has been shown that AD may affect systemic processes like glucose metabolism and cardiometabolic health and not only the central nervous system (Morris et al., 2014), thus prompting the search for an early biomarker of the disease in easily accessible tissues. The identification of such a diagnostic and therapeutic tool would meet the currently unmet and urgent need of early diagnosis of AD and easy implementation in the clinical practice.

The decreases expression of these four genes in both human iPSC-derived neurons and fibroblasts from AD patients suggest that they could play a role in different tissues and affect AD progression through regulating mitophagy and mitochondria homeostasis. Interestingly, in the case of fibroblasts these systematic changes in gene expression were more consistent in male individuals, suggesting the possibility of sex difference in their regulations and highlighting the importance of identifying the best model to mimic the changes observed in human samples.

## 5. Conclusion

Altogether, by optimising the use of public available resources from human AD brain studies, by combining different bioinformatic approaches and by narrowing the large-scale RNA-Seq data analysis to genes associated to mitophagy, this study highlights four genes (VCP, ARF1, GABARAPL1, and ACTB), as promising relevant genes in AD pathology. Although their changes were also further validated here in biological *in vitro* systems relevant for sporadic AD, such as human fibroblasts and iPSC-derived neurons, more functional experiments and *in vivo* studies are needed to confirm their value as potential novel biomarkers and targets for intervention.

## Data availability statement

The data presented in the study are deposited in the AD Knowledge Portal (https://adknowledgeportal.org), accession number syn9702085, and the GEO (https://www.ncbi.nlm.nih.gov/gds), accession number GSE110731. All data are previously published and publicly available.

## Author contributions

LJR, ZL, YL, TM, LB, and RA designed the study. TM and RA collected the datasets and performed the bioinformatic analysis. AO and YL performed the experiments and analysed the results. All authors contributed to writing the manuscript, reviewed the manuscript, and approved the submission for publication.
