# Supplementary material for: Pooled analysis of frontal lobe transcriptomic data identifies key mitophagy gene changes in Alzheimer's disease brain
Source: Front Aging Neurosci. 2023 Jun 9;15:1101216. doi: 10.3389/fnagi.2023.1101216 (PMC10288858; doi:10.3389/fnagi.2023.1101216)
Supplement: Supplementary file 1 [file Table_1.docx]

Supplementary Material

# Supplementary Figures and Tables

## Supplementary Tables

**Supplementary Table 1.** The information of primary skin fibroblasts

| Line | Age/sex | Clinical diagnosis |
| --- | --- | --- |
| AG02261 (Ctrl1) | 61/male | Non-affected |
| AG16086 (Ctrl2) | 67/female | Non-affected |
| AG07377 (AD1) | 60/male | Moderate Dementia |
| AG06263 (AD2) | 67/female | Moderate Dementia |
|  |  |  |

**Supplementary Table 2.** The information of iPSCs lines

|  | | |
| --- | --- | --- |
| Line | Age/sex | Clinical diagnosis |
| BPC927 | 73/female | Moderate Dementia |
| BPC404 | 74/female | Non-affected |
| BPC946 | 76/male | Moderate Dementia |
| BPC345 | 71/male | Non-affected |
| BPC937 | 79/female | Moderate Dementia |
| BPC406 | 78/female | Non-affected |

**Supplementary Table 3.** Candidate genes for experimental validation and their primers

| **Gene symbol** | **Gene name** | **Accesion number** | **Chromosome**  **location** | **Primer sequence**  **(5'-3')** | **Primer length** | **Amplicon size** | | **Tm** | | **%GC** | |
| --- | --- | --- | --- | --- | --- | --- | --- | --- | --- | --- | --- |
| ACTB | Actin beta | NM_001101.5 | chr7p22.1 | F: ACCAACTGGGACGACATGGA/ R: AGGTCTCAAACATGATCTGGGT | 20/22 | 151 | 60/57 | | 55/45 | |  |
| ARF1 | ADP ribosylation factor 1 | NM_001024226.2 | chr1q42.13 | F:GGTGGACAGCAATGACAGAGA/ R: TTGGGGAGGTCCTGCTTGTT | 20/20 | 123 | 59/61 | | 52/55 | |  |
| GABARAPL1 | GABA type A associated protein like 1 | NM_001363598.2 | chr12p13.2 | F:AGGGTCCCCGTGATTGTAGA / R: TAAGGCGTCCTCAGGTCTCA | 20/20 | 147 | 58/59 | | 55 | |  |
| GAPDH | Glyceraldehyde-3-phosphate dehydrogenase | NM_002046.7 | chr12p13.31 | F:TGTGGGCATCAATGGATTTGG  R:ACACCATGTATTCCGGGTCAAT | 21/22 | 116 | 52/53 | | 48/52 | |  |
| HPRT | Hypoxanthine Phosphoribosyltransferase 1 | NM_000194.4 | Xq26.2-q26.3 | F:CCTGGCGTCGTGATTAGTGAT R: AGACGTTCAGTCCTGTCCATAA | 21/22 | 131 | 62/61 | | 52/45 | |  |
| RPL13a | Ribosomal protein L13a | NM_001270491.2 | chr19q13.33 | F: CCACCGCCCTACGACAAGAAA/ R: TAGGCTTCAGACGCACGACC | 21/20 | 70 | 62 | | 57/60 | |  |
| TBP | TATA-box binding protein | NM_001172085.2 | chr6q27 | F:CCACGAACCACGGCACTGAT / R: TGGACTGTTCTTCACTCTTGGC | 20/22 | 73 | 63/60 | | 60/50 | |  |
| VCP | Valosin containing protein | NM_001354928.2 | chr9.p13.3 | F: GATGCCATCGCTCCCAAAAG/ R: CCCTGTCAAAGCGACCAAATC | 20/21 | 178 | 62 | | 55/52 | |  |
